# Crystal structure of 4-{[(naphthalen-2-yl)sulfonyl­amino]­meth­yl}cyclo­hexa­necarb­oxy­lic acid

**DOI:** 10.1107/S2056989015002054

**Published:** 2015-02-04

**Authors:** Muhammad Danish, Muhammad Nawaz Tahir, Asif Hussain, Muhammad Ashfaq, Muhammad Nadeem Sadiq

**Affiliations:** a Department of Chemistry, Institute of Natural Sciences, University of Gujrat, Gujrat 50700, Pakistan; b Department of Physics, University of Sargodha, Sargodha, Punjab, Pakistan

**Keywords:** crystal structure, sulfonamides, tranexamic acid, hydrogen bonding, C—H⋯π inter­actions

## Abstract

The title compound, C_18_H_21_NO_4_S, is a new sulfonamide derivative of tranexamic acid. In the crystal, mol­ecules form inversion dimers *via* O—H⋯O hydrogen bonds involving the carb­oxy­lic acid groups. Hydrogen bonding between the sulfonamide N—H group and the carb­oxy­lic acid O atom assembles the dimers into thick layers parallel to (100). The naphthalene groups of adjacent layers are arranged in a herring-bone motif. There are C—H⋯π inter­actions between the naphthalene rings of neighbouring layers.

## Related literature   

For related structures, see: Ashfaq *et al.* (2011*a* ▸,*b* ▸).
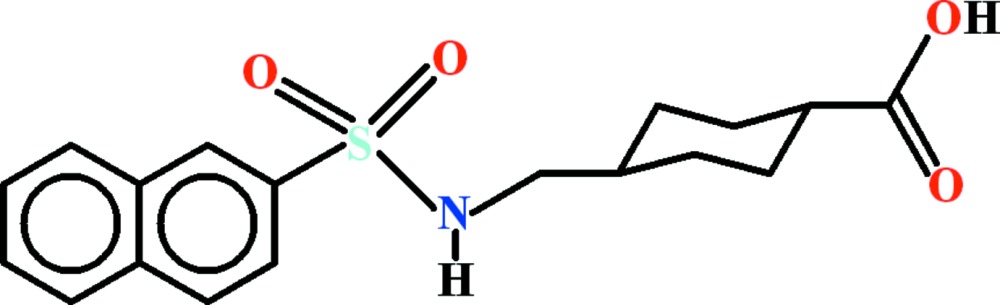


## Experimental   

### Crystal data  

C_18_H_21_NO_4_S
*M* *_r_* = 347.42
Monoclinic, 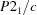

*a* = 16.5301 (13) Å
*b* = 6.0573 (4) Å
*c* = 17.0036 (12) Å
β = 100.810 (4)°
*V* = 1672.3 (2) Å^3^
*Z* = 4
Mo *K*α radiation
μ = 0.22 mm^−1^
*T* = 296 K
0.40 × 0.22 × 0.20 mm

### Data collection  

Bruker Kappa APEXII CCD diffractometer
Absorption correction: multi-scan (*SADABS*; Bruker, 2005 ▸) *T*_min_ = 0.920, *T*_max_ = 0.956
13364 measured reflections
3636 independent reflections
2315 reflections with *I* > 2σ(*I*)
*R*_int_ = 0.036

### Refinement  

*R*[*F* ^2^ > 2σ(*F* ^2^)] = 0.045
*wR*(*F* ^2^) = 0.117
*S* = 1.02
3636 reflections
218 parameters
H-atom parameters constrained
Δρ_max_ = 0.27 e Å^−3^
Δρ_min_ = −0.36 e Å^−3^

### 

Data collection: *APEX2* (Bruker, 2007 ▸); cell refinement: *SAINT* (Bruker, 2007 ▸); data reduction: *SAINT*; program(s) used to solve structure: *SHELXS97* (Sheldrick, 2008 ▸); program(s) used to refine structure: *SHELXL97* (Sheldrick, 2008 ▸); molecular graphics: *ORTEP-3 for Windows* (Farrugia, 2012 ▸) and *PLATON* (Spek, 2009 ▸); software used to prepare material for publication: *WinGX* (Farrugia, 2012 ▸) and *PLATON*.

## Supplementary Material

Crystal structure: contains datablock(s) global, I. DOI: 10.1107/S2056989015002054/gk2626sup1.cif

Structure factors: contains datablock(s) I. DOI: 10.1107/S2056989015002054/gk2626Isup2.hkl

Supporting information file. DOI: 10.1107/S2056989015002054/gk2626Isup3.cml

**Fig. 1.**
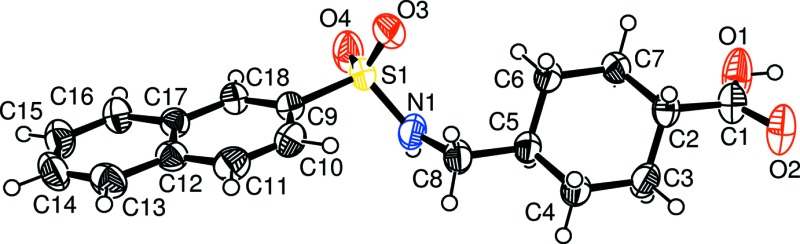
View of the title compound with the atom numbering scheme. Displacement ellipsoids are drawn at the 50% probability level. H-atoms are shown by small circles of arbitrary radii.

. DOI: 10.1107/S2056989015002054/gk2626fig1.tif
View of the title compound with the atom numbering scheme. Displacement ellipsoids are drawn at the 50% probability level. H-atoms are shown by small circles of arbitrary radii.

**Fig. 2.**
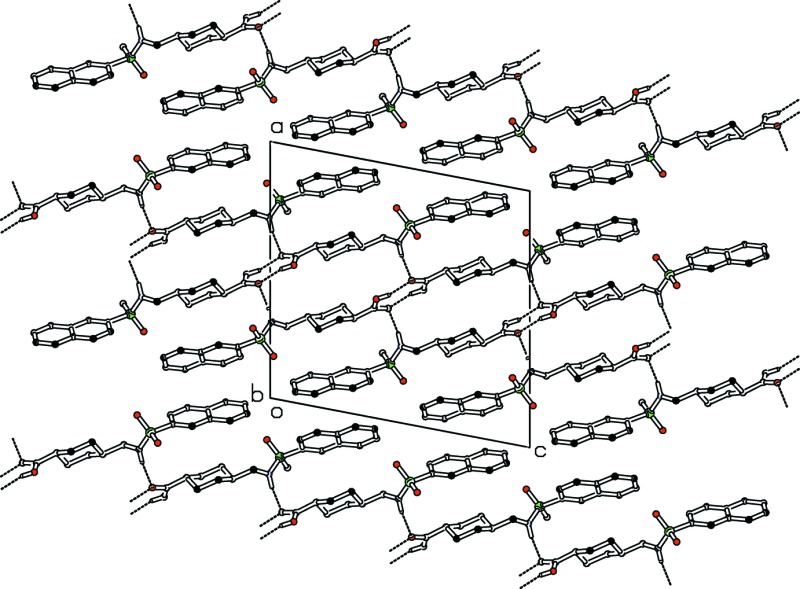
View of the crystal packing in the title compound with hydrogen bonds shown as dashed lines.

. DOI: 10.1107/S2056989015002054/gk2626fig2.tif
View of the crystal packing in the title compound with hydrogen bonds shown as dashed lines.

CCDC reference: 1046512

Additional supporting information:  crystallographic information; 3D view; checkCIF report

## Figures and Tables

**Table 1:** Hydrogen-bond geometry (, ) *Cg*1 and *Cg*2 are the centroids of the C9C12/C17/C18 and C12C17 rings, respectively.

*D*H*A*	*D*H	H*A*	*D* *A*	*D*H*A*
O1H1O2^i^	0.82	1.83	2.623(2)	163
N1H1*A*O2^ii^	0.86	2.46	3.043(2)	124
C11H11*Cg*1^iii^	0.93	2.91	3.639(3)	137
C13H13*Cg*2^iii^	0.93	2.82	3.527(3)	134

Symmetry codes: (i) 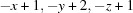; (ii) 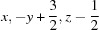; (iii) 
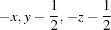
.

## supplementary crystallographic information

### S1. Structural commentary

The title compound (Fig. 1), a derivative of tranexamic acid, was prepared as a part of our studies on sulfonamides. It is also planned to use this compound for complexing metal ions. The crystal structures of similar compounds have been reported earlier [4-((((4-methyl­phenyl)­sulfonyl)­amino)­methyl)­cyclo­hexane­carb­oxy­lic acid (Ashfaq *et al.*, 2011*a*); 4-((4-meth­oxy­benzene­sulfonamido)­methyl)­cyclo­hexane-1- carb­oxy­lic acid (Ashfaq *et al.*, 2011*b*).

The cyclo­hexyl ring of tranexamic acidic moiety is in a chair form. The atoms forming the basal plane *A* (C3/C4/C6/C7) are co-planar (r. m.s deviation of 0.0032 Å). The C2 and C5 atoms are at a distance of 0.653 (3) and 0.678 (3) Å from the plane *A*. The naphthalene ring *B* (C9—C18) is also planar with r. m.s deviation of 0.0051 Å. The dihedral angle between A/B is 45.76 (6)°. The carb­oxy­lic group is oriented at a dihedral angle of 38.5 (2)° with the plane *A*. The molecules form centrosymmetric dimers due to O—H···O type hydrogen bonds of the carb­oxy­lic groups and complete *R*_2_^2^(8) ring motif. The dimers are inter­linked through N—H···O bonds (Table 1, Fig. 2) completing *R*_2_^2^(22) ring motif to form two dimensional polymeric network parallel to (100). There are C—H···π (Table 1) inter­actions between the naphthalene rings from neighboring (100) layers.

### S2. Synthesis and crystallization

The title compound was prepared from 2-naphthalene­sulfonyl chloride (1 mmol, 0.226 g) and tranexamic acid(1 mmol, 0.157 g) added to 20 ml of distilled water. The mixture was constantly stirred and its pH was maintained at 8–9 by using 1 *M* sodium bicarbonate solution. Completion of the reaction after 3 h was confirmed by observing clear solution. The final product was precipitated by adding 0.1 *M* HCl solution, separated and recrystallized from ethanol-water mixture in 1:1 volume ratio. Colorless needles suitable for X-ray data collection were obtained after one week (yield: 59%, m.p. 483 K).

### S3. Refinement

Crystal data, data collection and structure refinement details are summarized in Table 1. The H-atoms were positioned geometrically (C–H = 0.93—0.98 Å, N–H = 0.86 Å, O–H = 0.82 Å) and refined as riding with *U*_iso_(H) = *xU*_eq_ (C, N, O), where *x* = 1.5 for hy­droxy and *x* = 1.2 for all other H-atoms.

### Crystal data

**Table:**

C_18_H_21_NO_4_S	*F*(000) = 736
*M**_r_* = 347.42	*D*_x_ = 1.380 Mg m^−^^3^
Monoclinic, *P*2_1_/*c*	Mo *K*α radiation, λ = 0.71073 Å
*a* = 16.5301 (13) Å	Cell parameters from 2315 reflections
*b* = 6.0573 (4) Å	θ = 1.3–27.0°
*c* = 17.0036 (12) Å	µ = 0.22 mm^−^^1^
β = 100.810 (4)°	*T* = 296 K
*V* = 1672.3 (2) Å^3^	Needle, colourless
*Z* = 4	0.40 × 0.22 × 0.20 mm

### Data collection

**Table:**

Bruker Kappa APEXII CCD diffractometer	3636 independent reflections
Radiation source: fine-focus sealed tube	2315 reflections with *I* > 2σ(*I*)
Graphite monochromator	*R*_int_ = 0.036
Detector resolution: 7.80 pixels mm^-1^	θ_max_ = 27.0°, θ_min_ = 1.3°
ω scans	*h* = −21→20
Absorption correction: multi-scan (*SADABS*; Bruker, 2005)	*k* = −7→6
*T*_min_ = 0.920, *T*_max_ = 0.956	*l* = −21→21
13364 measured reflections	

### Refinement

**Table:**

Refinement on *F*^2^	Primary atom site location: structure-invariant direct methods
Least-squares matrix: full	Secondary atom site location: difference Fourier map
*R*[*F*^2^ > 2σ(*F*^2^)] = 0.045	Hydrogen site location: inferred from neighbouring sites
*wR*(*F*^2^) = 0.117	H-atom parameters constrained
*S* = 1.02	*w* = 1/[σ^2^(*F*_o_^2^) + (0.049*P*)^2^ + 0.3363*P*] where *P* = (*F*_o_^2^ + 2*F*_c_^2^)/3
3636 reflections	(Δ/σ)_max_ < 0.001
218 parameters	Δρ_max_ = 0.27 e Å^−^^3^
0 restraints	Δρ_min_ = −0.36 e Å^−^^3^

### Special details

**Table:**

Geometry. All e.s.d.'s (except the e.s.d. in the dihedral angle between two l.s. planes) are estimated using the full covariance matrix. The cell e.s.d.'s are taken into account individually in the estimation of e.s.d.'s in distances, angles and torsion angles; correlations between e.s.d.'s in cell parameters are only used when they are defined by crystal symmetry. An approximate (isotropic) treatment of cell e.s.d.'s is used for estimating e.s.d.'s involving l.s. planes.
Refinement. Refinement of *F*^2^ against ALL reflections. The weighted *R*-factor *wR* and goodness of fit *S* are based on *F*^2^, conventional *R*-factors *R* are based on *F*, with *F* set to zero for negative *F*^2^. The threshold expression of *F*^2^ > σ(*F*^2^) is used only for calculating *R*-factors(gt) *etc*. and is not relevant to the choice of reflections for refinement. *R*-factors based on *F*^2^ are statistically about twice as large as those based on *F*, and *R*-factors based on ALL data will be even larger.

### Fractional atomic coordinates and isotropic or equivalent isotropic displacement parameters (Å^2^)

**Table:**

	*x*	*y*	*z*	*U*_iso_*/*U*_eq_	
S1	0.22360 (4)	1.11590 (10)	−0.03913 (3)	0.04790 (19)	
O1	0.46462 (13)	1.1212 (3)	0.40940 (10)	0.0784 (6)	
H1	0.4886	1.1331	0.4560	0.118*	
O2	0.43679 (11)	0.7932 (3)	0.45431 (9)	0.0664 (5)	
O3	0.16554 (11)	1.1324 (3)	0.01273 (9)	0.0690 (5)	
O4	0.25871 (12)	1.3099 (3)	−0.06651 (9)	0.0676 (5)	
N1	0.29769 (11)	0.9664 (3)	0.00539 (10)	0.0494 (5)	
H1A	0.3455	0.9895	−0.0061	0.059*	
C1	0.42778 (14)	0.9398 (4)	0.40022 (12)	0.0481 (6)	
C2	0.37262 (13)	0.8893 (3)	0.32200 (12)	0.0407 (5)	
H2	0.3195	0.8392	0.3331	0.049*	
C3	0.40971 (15)	0.7018 (4)	0.28063 (13)	0.0513 (6)	
H3A	0.4646	0.7433	0.2737	0.062*	
H3B	0.4146	0.5715	0.3144	0.062*	
C4	0.35752 (15)	0.6468 (4)	0.19930 (12)	0.0493 (6)	
H4A	0.3848	0.5332	0.1737	0.059*	
H4B	0.3047	0.5891	0.2067	0.059*	
C5	0.34370 (13)	0.8489 (3)	0.14548 (12)	0.0404 (5)	
H5	0.3975	0.9008	0.1370	0.048*	
C6	0.30492 (14)	1.0314 (4)	0.18683 (11)	0.0436 (5)	
H6A	0.2508	0.9847	0.1944	0.052*	
H6B	0.2980	1.1615	0.1530	0.052*	
C7	0.35715 (14)	1.0900 (3)	0.26754 (12)	0.0440 (5)	
H7A	0.3293	1.2029	0.2930	0.053*	
H7B	0.4094	1.1498	0.2596	0.053*	
C8	0.29161 (15)	0.7938 (4)	0.06398 (12)	0.0484 (6)	
H8A	0.3099	0.6546	0.0452	0.058*	
H8B	0.2345	0.7769	0.0693	0.058*	
C9	0.17432 (12)	0.9711 (4)	−0.12510 (11)	0.0388 (5)	
C10	0.14017 (14)	0.7621 (4)	−0.11635 (13)	0.0484 (6)	
H10	0.1453	0.6988	−0.0658	0.058*	
C11	0.09970 (14)	0.6527 (4)	−0.18159 (13)	0.0483 (6)	
H11	0.0769	0.5149	−0.1753	0.058*	
C12	0.09160 (12)	0.7449 (4)	−0.25889 (12)	0.0405 (5)	
C13	0.04936 (14)	0.6359 (4)	−0.32851 (14)	0.0514 (6)	
H13	0.0258	0.4982	−0.3240	0.062*	
C14	0.04315 (15)	0.7311 (4)	−0.40141 (14)	0.0577 (7)	
H14	0.0150	0.6586	−0.4466	0.069*	
C15	0.07844 (14)	0.9367 (4)	−0.40943 (13)	0.0546 (6)	
H15	0.0742	0.9988	−0.4600	0.066*	
C16	0.11865 (13)	1.0470 (4)	−0.34486 (12)	0.0465 (6)	
H16	0.1413	1.1849	−0.3513	0.056*	
C17	0.12663 (12)	0.9548 (3)	−0.26764 (11)	0.0379 (5)	
C18	0.16767 (12)	1.0656 (4)	−0.19883 (11)	0.0389 (5)	
H18	0.1904	1.2042	−0.2037	0.047*	

### Atomic displacement parameters (Å^2^)

**Table:**

	*U*^11^	*U*^22^	*U*^33^	*U*^12^	*U*^13^	*U*^23^
S1	0.0589 (4)	0.0505 (4)	0.0310 (3)	0.0083 (3)	0.0001 (2)	−0.0049 (2)
O1	0.0973 (15)	0.0765 (13)	0.0477 (10)	−0.0415 (11)	−0.0217 (10)	0.0108 (9)
O2	0.0784 (13)	0.0717 (12)	0.0406 (9)	−0.0267 (9)	−0.0110 (8)	0.0139 (8)
O3	0.0673 (12)	0.0963 (14)	0.0427 (9)	0.0280 (10)	0.0083 (8)	−0.0136 (9)
O4	0.1019 (15)	0.0441 (11)	0.0472 (9)	−0.0130 (9)	−0.0105 (9)	−0.0006 (8)
N1	0.0459 (11)	0.0638 (13)	0.0364 (10)	0.0060 (9)	0.0022 (8)	0.0065 (9)
C1	0.0510 (14)	0.0551 (16)	0.0358 (12)	−0.0152 (11)	0.0021 (10)	0.0050 (11)
C2	0.0390 (12)	0.0473 (14)	0.0335 (10)	−0.0092 (10)	0.0007 (9)	0.0025 (9)
C3	0.0609 (16)	0.0422 (14)	0.0439 (12)	0.0037 (11)	−0.0079 (11)	0.0061 (10)
C4	0.0592 (15)	0.0416 (14)	0.0416 (12)	0.0057 (11)	−0.0050 (11)	−0.0024 (10)
C5	0.0429 (13)	0.0418 (13)	0.0338 (10)	0.0015 (10)	0.0006 (9)	0.0004 (9)
C6	0.0484 (13)	0.0421 (13)	0.0366 (11)	0.0051 (10)	−0.0015 (10)	0.0008 (10)
C7	0.0519 (14)	0.0405 (13)	0.0375 (11)	0.0018 (10)	0.0027 (10)	−0.0035 (9)
C8	0.0590 (15)	0.0462 (15)	0.0357 (11)	0.0009 (11)	−0.0019 (10)	−0.0015 (10)
C9	0.0398 (12)	0.0422 (13)	0.0337 (11)	0.0049 (9)	0.0048 (9)	−0.0018 (9)
C10	0.0567 (15)	0.0465 (14)	0.0408 (12)	0.0049 (11)	0.0062 (11)	0.0099 (11)
C11	0.0517 (14)	0.0387 (14)	0.0546 (14)	−0.0024 (10)	0.0102 (11)	0.0059 (11)
C12	0.0355 (12)	0.0427 (13)	0.0437 (12)	0.0011 (10)	0.0083 (9)	−0.0036 (10)
C13	0.0475 (14)	0.0488 (15)	0.0576 (15)	−0.0080 (11)	0.0087 (11)	−0.0147 (12)
C14	0.0552 (16)	0.0710 (18)	0.0440 (13)	−0.0046 (13)	0.0022 (12)	−0.0222 (12)
C15	0.0532 (15)	0.0743 (19)	0.0362 (12)	−0.0012 (13)	0.0079 (11)	−0.0049 (11)
C16	0.0507 (14)	0.0538 (15)	0.0349 (11)	−0.0045 (11)	0.0076 (10)	0.0005 (10)
C17	0.0366 (12)	0.0407 (13)	0.0367 (11)	0.0007 (9)	0.0074 (9)	−0.0023 (9)
C18	0.0395 (12)	0.0412 (13)	0.0351 (11)	−0.0033 (9)	0.0049 (9)	−0.0010 (9)

### Geometric parameters (Å, º)

**Table:**

S1—O3	1.4235 (17)	C6—H6B	0.9700
S1—O4	1.4267 (17)	C7—H7A	0.9700
S1—N1	1.5955 (19)	C7—H7B	0.9700
S1—C9	1.767 (2)	C8—H8A	0.9700
O1—C1	1.252 (3)	C8—H8B	0.9700
O1—H1	0.8200	C9—C18	1.364 (3)
O2—C1	1.267 (3)	C9—C10	1.405 (3)
N1—C8	1.461 (3)	C10—C11	1.356 (3)
N1—H1A	0.8600	C10—H10	0.9300
C1—C2	1.496 (3)	C11—C12	1.411 (3)
C2—C7	1.521 (3)	C11—H11	0.9300
C2—C3	1.524 (3)	C12—C17	1.416 (3)
C2—H2	0.9800	C12—C13	1.420 (3)
C3—C4	1.523 (3)	C13—C14	1.353 (3)
C3—H3A	0.9700	C13—H13	0.9300
C3—H3B	0.9700	C14—C15	1.392 (3)
C4—C5	1.520 (3)	C14—H14	0.9300
C4—H4A	0.9700	C15—C16	1.349 (3)
C4—H4B	0.9700	C15—H15	0.9300
C5—C6	1.515 (3)	C16—C17	1.410 (3)
C5—C8	1.525 (3)	C16—H16	0.9300
C5—H5	0.9800	C17—C18	1.408 (3)
C6—C7	1.520 (3)	C18—H18	0.9300
C6—H6A	0.9700		
			
O3—S1—O4	120.48 (11)	C6—C7—C2	111.43 (17)
O3—S1—N1	107.01 (10)	C6—C7—H7A	109.3
O4—S1—N1	107.41 (11)	C2—C7—H7A	109.3
O3—S1—C9	106.71 (10)	C6—C7—H7B	109.3
O4—S1—C9	106.94 (10)	C2—C7—H7B	109.3
N1—S1—C9	107.73 (10)	H7A—C7—H7B	108.0
C1—O1—H1	109.5	N1—C8—C5	111.32 (18)
C8—N1—S1	125.80 (16)	N1—C8—H8A	109.4
C8—N1—H1A	117.1	C5—C8—H8A	109.4
S1—N1—H1A	117.1	N1—C8—H8B	109.4
O1—C1—O2	122.5 (2)	C5—C8—H8B	109.4
O1—C1—C2	119.4 (2)	H8A—C8—H8B	108.0
O2—C1—C2	118.1 (2)	C18—C9—C10	120.71 (19)
C1—C2—C7	112.50 (18)	C18—C9—S1	119.82 (17)
C1—C2—C3	109.38 (18)	C10—C9—S1	119.44 (16)
C7—C2—C3	110.55 (16)	C11—C10—C9	120.0 (2)
C1—C2—H2	108.1	C11—C10—H10	120.0
C7—C2—H2	108.1	C9—C10—H10	120.0
C3—C2—H2	108.1	C10—C11—C12	121.0 (2)
C4—C3—C2	111.94 (18)	C10—C11—H11	119.5
C4—C3—H3A	109.2	C12—C11—H11	119.5
C2—C3—H3A	109.2	C11—C12—C17	118.95 (19)
C4—C3—H3B	109.2	C11—C12—C13	122.6 (2)
C2—C3—H3B	109.2	C17—C12—C13	118.5 (2)
H3A—C3—H3B	107.9	C14—C13—C12	120.4 (2)
C5—C4—C3	111.48 (18)	C14—C13—H13	119.8
C5—C4—H4A	109.3	C12—C13—H13	119.8
C3—C4—H4A	109.3	C13—C14—C15	120.6 (2)
C5—C4—H4B	109.3	C13—C14—H14	119.7
C3—C4—H4B	109.3	C15—C14—H14	119.7
H4A—C4—H4B	108.0	C16—C15—C14	121.0 (2)
C6—C5—C4	109.71 (17)	C16—C15—H15	119.5
C6—C5—C8	111.43 (18)	C14—C15—H15	119.5
C4—C5—C8	111.49 (17)	C15—C16—C17	120.5 (2)
C6—C5—H5	108.0	C15—C16—H16	119.8
C4—C5—H5	108.0	C17—C16—H16	119.8
C8—C5—H5	108.0	C18—C17—C16	122.1 (2)
C5—C6—C7	111.78 (17)	C18—C17—C12	118.91 (18)
C5—C6—H6A	109.3	C16—C17—C12	119.00 (19)
C7—C6—H6A	109.3	C9—C18—C17	120.5 (2)
C5—C6—H6B	109.3	C9—C18—H18	119.8
C7—C6—H6B	109.3	C17—C18—H18	119.8
H6A—C6—H6B	107.9		
			
O3—S1—N1—C8	28.1 (2)	O3—S1—C9—C10	−55.32 (19)
O4—S1—N1—C8	158.77 (17)	O4—S1—C9—C10	174.51 (17)
C9—S1—N1—C8	−86.34 (19)	N1—S1—C9—C10	59.31 (19)
O1—C1—C2—C7	−10.3 (3)	C18—C9—C10—C11	−0.4 (3)
O2—C1—C2—C7	171.3 (2)	S1—C9—C10—C11	177.71 (17)
O1—C1—C2—C3	112.9 (3)	C9—C10—C11—C12	0.4 (3)
O2—C1—C2—C3	−65.4 (3)	C10—C11—C12—C17	0.1 (3)
C1—C2—C3—C4	−178.34 (18)	C10—C11—C12—C13	−179.7 (2)
C7—C2—C3—C4	−53.9 (3)	C11—C12—C13—C14	180.0 (2)
C2—C3—C4—C5	55.6 (3)	C17—C12—C13—C14	0.2 (3)
C3—C4—C5—C6	−56.2 (3)	C12—C13—C14—C15	0.4 (4)
C3—C4—C5—C8	179.88 (19)	C13—C14—C15—C16	−0.9 (4)
C4—C5—C6—C7	57.0 (2)	C14—C15—C16—C17	0.7 (4)
C8—C5—C6—C7	−179.07 (18)	C15—C16—C17—C18	−179.6 (2)
C5—C6—C7—C2	−56.8 (2)	C15—C16—C17—C12	−0.2 (3)
C1—C2—C7—C6	176.83 (18)	C11—C12—C17—C18	−0.6 (3)
C3—C2—C7—C6	54.2 (2)	C13—C12—C17—C18	179.13 (19)
S1—N1—C8—C5	−117.30 (19)	C11—C12—C17—C16	179.93 (19)
C6—C5—C8—N1	73.3 (2)	C13—C12—C17—C16	−0.3 (3)
C4—C5—C8—N1	−163.77 (18)	C10—C9—C18—C17	−0.2 (3)
O3—S1—C9—C18	122.76 (18)	S1—C9—C18—C17	−178.28 (15)
O4—S1—C9—C18	−7.4 (2)	C16—C17—C18—C9	−179.9 (2)
N1—S1—C9—C18	−122.61 (17)	C12—C17—C18—C9	0.7 (3)

### Hydrogen-bond geometry (Å, º)

Cg1 and Cg2 are the centroids of the C9–C12/C17/C18 and C12–C17 rings, respectively.

**Table:**

*D*—H···*A*	*D*—H	H···*A*	*D*···*A*	*D*—H···*A*
O1—H1···O2^i^	0.82	1.83	2.623 (2)	163
N1—H1*A*···O2^ii^	0.86	2.46	3.043 (2)	124
C11—H11···*Cg*1^iii^	0.93	2.91	3.639 (3)	137
C13—H13···*Cg*2^iii^	0.93	2.82	3.527 (3)	134

Symmetry codes: (i) −*x*+1, −*y*+2, −*z*+1; (ii) *x*, −*y*+3/2, *z*−1/2; (iii) −*x*, *y*−1/2, −*z*−1/2.
