# Supplementary figures and images for: Crystal structure of 4-{[(naphthalen-2-yl)sulfonyl­amino]­meth­yl}cyclo­hexa­necarb­oxy­lic acid
Source: Acta Crystallogr E Crystallogr Commun. 2015 Feb 4;71(Pt 3):o145. doi: 10.1107/S2056989015002054 (PMC4350692; doi:10.1107/S2056989015002054)

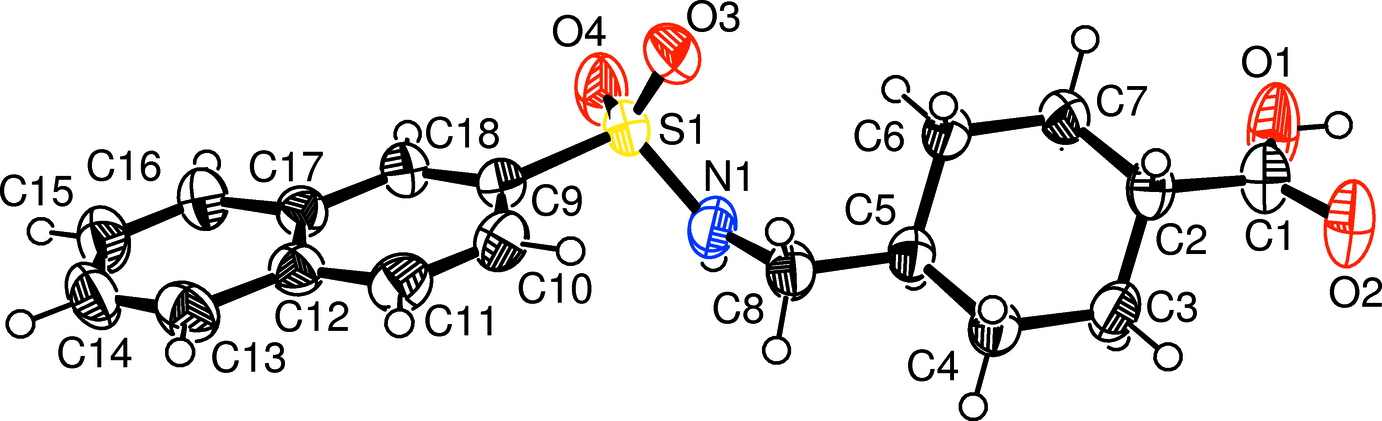

Supplement: Supplementary file 4 [file e-71-0o145-fig1.tif]

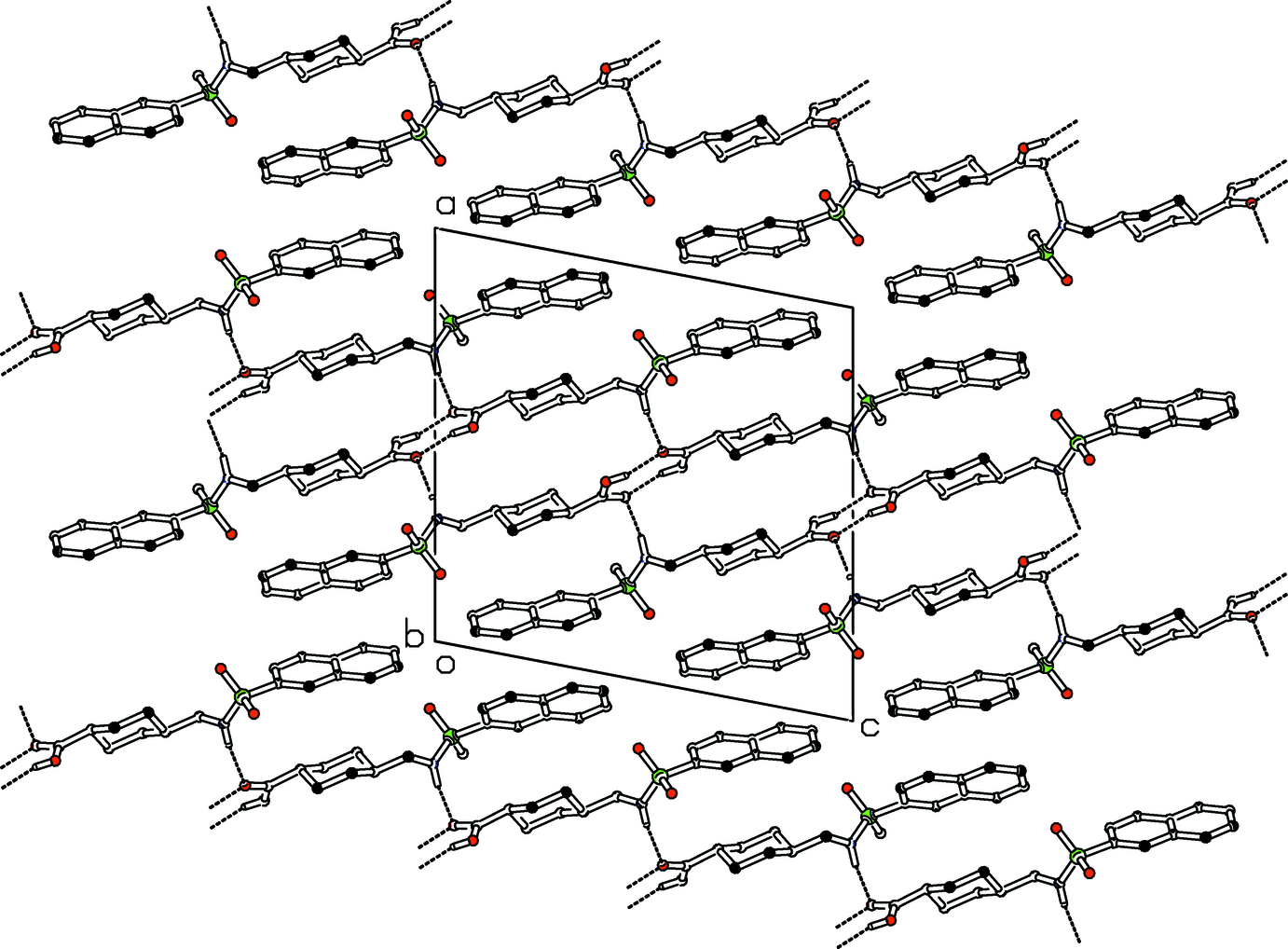

Supplement: Supplementary file 5 [file e-71-0o145-fig2.tif]
